# Knowledge and Attitudes Toward Organ Donation and Transplantation Among Nursing Students: A Multicentre Cross-Sectional Study

**DOI:** 10.3390/nursrep15060181

**Published:** 2025-05-22

**Authors:** Luca Bertocchi, Cristina Petrucci, Massimo Alex Calzetta, Angelo Dante, Felice Curcio, Loreto Lancia, Cesar Ivan Aviles Gonzalez

**Affiliations:** 1 Haematology Unit, Trieste University Hospital (ASUGI), Piazza dell’Ospitale 1, 34125 Trieste, Italy; luca.bertocchi@asugi.sanita.fvg.it; 2 Department of Health, Life and Environment Sciences, University of L’Aquila, 67100 L’Aquila, Italy; cristina.petrucci@univaq.it (C.P.); angelo.dante@univaq.it (A.D.); loreto.lancia@univaq.it (L.L.); 3 Clinical Medicine, Santa Maria della Misericordia Hospital of Udine, Piazzale Santa Maria della Misericordia 15, 33100 Udine, Italy; massimoalex.calzetta@asufc.sanita.fvg.it; 4 Faculty of Medicine and Surgery, University of Sassari (UNISS), 07100 Sassari, Italy; 5 Department of Nursing, University of Studies of Enna Kore, 94100 Enna, Italy

**Keywords:** education, knowledge, student attitude, nursing, organ donation and transplantation

## Abstract

**Background/Objectives**: Organ transplantation is a vital treatment for individuals with advanced chronic-degenerative diseases. However, the global shortage of donated organs remains a significant challenge. Improving knowledge and attitudes could positively impact this issue. This study assessed the knowledge and attitudes of nursing students regarding organ donation and transplantation. **Methods**: A cross-sectional study was conducted using a previously validated questionnaire administered to 235 second- and third-year undergraduate nursing students from two Italian universities. **Results**: The response rate was 67.3%. Only 40.4% of students felt adequately informed about transplants and brain death, while 12.8% would not authorise organ transplantation for a family member. Willingness to authorise organ procurement from family members in a brain-dead state was positively associated with being atheist or agnostic (χ^2^ = 7.235; *p* = 0.022), being in the third year of study (χ^2^ = 4.282; *p* = 0.039) and having positive self-assessed knowledge (χ^2^ = 8.061; *p* = 0.005). **Conclusions**: Nursing students exhibited suboptimal knowledge and positive attitudes toward organ and tissue donation. However, there is a need for health policymakers to implement strategies to raise awareness of the importance of organ donation through school and community programmes and public education campaigns.

## 1. Introduction

Organ donation and transplantation offer life-saving therapies for individuals with advanced stages of chronic, irreversible and degenerative diseases [1,2,3]. The scientific community and legal systems in numerous countries acknowledge brain death as the cessation of a person’s vital functions. Embracing this concept has played a pivotal role in advancing the organ donation and transplantation process, particularly because a substantial portion of organ donors are individuals who have experienced brain death [4,5].

Over the years, Italy has seen a systematic increase in the number of donors and transplants. While, from 2001 to 2011, the number of donors and transplants had percentage increases of 25% and 14%, respectively, from 2012 to 2022, this growth had a further boost, reaching a 38% increase for donations and a 25% increase for transplants. In 2022, donors (deceased and living) and resulting transplants were 1.830 and 3.879, respectively [6]. However, the global shortage of donated organs remains a critical issue, as it fails to meet the transplant demands of patients on waiting lists [7,8,9]. To address this challenge, it is crucial to adopt strategies that focus on increasing self-awareness and understanding about organ donation, transplantation and brain death. As a result, solid knowledge and clinical skills concerning organ donation, transplant and brain death are important elements for guaranteeing better teamwork and approaches toward patients.

Nurses play a pivotal role in influencing potential donors’ decisions, as they are often the first point of contact with patients and their families. Nurses can collaborate with other team members [10] to implement effective strategies to encourage more organ donations. Promising approaches to increase donation rates are educational programs for patients and family members [1], revisions to nursing curricula [11,12], counselling for patients and families [10] and the inclusion of a nurse specialist in the organ donation [13].

In nursing education, incorporating comprehensive organ donation content into curricula is crucial. This integration ensures that future nurses are well prepared to engage in discussions about organ donation and transplantation, thereby fostering a more informed healthcare workforce as nursing students represent the future workforce of clinical professionals [14,15]. However, studies have shown that the knowledge and attitudes toward organ donation among nursing students often do not meet the desired standards [10,16,17]. Several factors contribute to this, including the lack of seminars or courses on organ donation in academic curricula [1,18,19,20,21], as well as cultural aspects of organ donation in the country [1,22,23,24].

The lack of knowledge about organ donation by health professionals poses significant challenges at both the healthcare and educational levels. This gap in knowledge contributes to fewer transplants and a growing number of individuals being on transplant waiting lists due to the failure to recognise potential donors [25]. Moreover, the absence of adequate knowledge transmission during training can negatively impact nurses’ ability to collaborate with multidisciplinary teams and support potential donors and their caregivers. To address this issue, early exposure to donation information during nursing university courses has the potential to improve future nurses’ clinical practice, enhancing their ability to collaborate effectively within the healthcare team and fostering more positive attitudes toward organ donation [26]. Increasing students’ knowledge and cultivating positive attitudes toward organ donation can be achieved through dedicated lessons integrated into their study path. Additionally, their willingness to donate organs may also be influenced by a combination of environmental, socio-cultural and educational variables [27,28,29]. In this regard, lectures and reading materials related to the topic have demonstrated a favourable impact on improving the knowledge and attitudes of nursing students [21,30,31,32,33,34].

Although there have been previous studies investigating knowledge and attitudes about organ donation [1,11,27,35,36], to the best of the authors’ knowledge, there is a paucity of multicentre studies in Italy investigating nursing students’ knowledge and attitudes about organ donation and transplantation. Thus, this study aims to assess the knowledge and attitudes of nursing students regarding organ donation and transplantation while comparing these aspects between second- and third-year nursing students.

## 2. Materials and Methods

### 2.1. Study Design and Setting

A cross-sectional study was conducted on a non-probabilistic sample of students enrolled in undergraduate courses at two Italian universities. The Strengthening the Reporting of Observational Studies in Epidemiology (STROBE) checklist was used for transparency in the reporting of the study [37].

This study was conducted in the Bachelor’s Degree programs in Nursing at the University of Trieste and the University of L’Aquila, Italy. The study plan for nursing degrees in Italy is three years long and requires the completion of 180 European Credit Transfer System (ECTS) credits, 60 of which are to be acquired in internship training activities. The degree program is structured to integrate theoretical learning with practical clinical experience.

### 2.2. Participants and Sampling Procedure

A non-probabilistic convenience sampling approach was adopted, inviting all eligible second- and third-year undergraduate nursing students enrolled at the University of Trieste and the University of L’Aquila to participate voluntarily in this study. The total eligible population consisted of 349 students, who were contacted via the university’s e-mail system with a link to the online questionnaire. Data collection took place from 8 to 28 October 2021.

### 2.3. Inclusion and Exclusion Criteria and Sample Size Estimation

Participants eligible for inclusion were second- or third-year nursing students who received the survey invitation via their university e-mail. First-year students were excluded because they had not yet acquired any theoretical knowledge or practical experience of transplantation and organ and tissue donation; these topics are only introduced in the curriculum from the second year onward.

The minimum required sample size was calculated using G*Power (Version 3.1.9.7 © Heinrich-Heine-Universität Düsseldorf) based on an a priori power analysis for chi-square tests (goodness-of-fit, contingency tables). Assuming a medium effect size (w = 0.30), a significance level of α = 0.05 and a power (1-*β*) of 0.80, the analysis indicated that a minimum of 183 participants was required to detect significant differences. The final sample of 235 students, therefore, exceeded the minimum required sample size.

### 2.4. Instrument and Study Variables

The knowledge and attitudes of university nursing students toward organ donation were assessed using a self-administered questionnaire. Socio-demographic characteristics, including age, sex, high school attended, religion, university site, academic year and student status, were also collected.

The organ donation questionnaire, used with permission from the original author, was originally developed and validated by Giusti and colleagues (2015) through a literature review and pilot testing, assessing both content and face validity [27]. The instrument is divided in five main sections: (1) self-assessment of knowledge (e.g., Do you think to have sufficient information about transplantation and brain death?); (2) scientific knowledge (e.g., what is ‘brain death’?); (3) attitudes, experiences and personal opinions on donation, organ procurement and transplantation (e.g., Do you believe in the effectiveness of transplants?); (4) experience and lived experiences; (5) information and proposals [27]. As the tool includes mixed-format items (e.g., correct/incorrect knowledge questions, dichotomous attitude items), no psychometric analyses such as internal consistency or test–retest reliability were conducted in either the original study or in the present research.

### 2.5. Bias

To minimise the response bias and social desirability bias, the anonymity and confidentiality of the responses were guaranteed to encourage honest answers and prevent any pressure that might arise from disclosing personal beliefs. To ensure that the sample of nursing students was representative of a broader nursing student population and minimise sample representation bias, the sample included students from two Italian universities. Both universities do not offer specific courses on organ donation and transplantation, although these topics are generally covered during the second and third year.

### 2.6. Statistical Analysis

Continuous variables were described by central tendency (mean, median) and dispersion measures such as standard deviation (SD) or interquartile range (IQR). Categorical variables were described as numbers (*n*) and percentages (%). The normality in distribution of continuous variables was visually assessed using histograms, boxplots, and Q-Q plots and verified using the Kolmogorov–Smirnov test. For categorical variables, the comparisons between groups were performed using the χ^2^ test, while for continuous variables, the Mann–Whitney U test was adopted. The level of statistical significance was set at *p* < 0.05. All data were analysed using the IBM SPSS version 28.0 (IBM Corp., Armonk, NY, USA).

### 2.7. Ethical Considerations

This study was conducted according to the ethical principles stated by the Declaration of Helsinki and its later amendments and with the General Data Protection Regulation (EU) 2016/679 (GDPR). Approval was granted by the Presidency Boards of the Bachelor’s Degree Programs in Nursing at both universities, with the formal authorisation dated 28 August 2020.

Consent for inclusion and data treatment was acquired in electronic format; only after giving informed consent by clicking yes was it possible to start the questionnaire. Students were informed that their participation was voluntary and anonymous, according to Italian Data protection law (e.g., Decree n. 196/2003). In addition, students were informed that they could leave the study at any time without any adverse consequences for their university program.

## 3. Results

### 3.1. Socio-Demographic Characteristics

A total of 235 second- and third-year nursing students completed the questionnaires with an overall response rate of 67.3%. The mean age of the students was 23.5 years (SD 4.83), and they were mostly female (84.7%). The most prevalent upper-secondary school attended was scientific (37.5%). The majority of students were Catholic (59.2%), 38.3% were atheist or agnostic and the remaining 8.1% were Orthodox, Muslim, Protestant or Hindu. Most of the students attended the third year (57.3%), and more than 95% of them were ‘in progress’ with their academic studies (Table 1).

### 3.2. Knowledge About Organ Donation, Transplantation and Brain Death

In the self-rated question about knowledge on transplants and brain death, only 40.4% of the students believe that they have enough information about these topics. Stratifying by academic year, a statistically significant greater percentage of third-year students believed themselves to have enough information on these compared to second-year students (*p* = 0.011); almost all the participants (97.9%) correctly stated the presence of laws in Italy regulating organ donation. Almost a quarter of the students did not provide the correct definition of brain death. There was a high awareness of the difference between ‘brain death’ and ‘cardiac death’, with 98.3% of students giving correct answers, although almost 12% of the sample believed that it is possible to return to life after being diagnosed with brain death. Moreover, 21.7% of students were unaware of the greater workload required for a patient in a brain death state, although there was a statistically significant higher percentage of correct answers in third-year students in comparison to second-year students (*p* = 0.008). Finally, 22.5% of the sample wrongly claimed that there are no interferences between drugs, toxic and narcotic substances and the clinical conditions for the diagnosis of brain death (Table 2).

### 3.3. Attitude Toward Organ Donation

It appears that almost all students (99.6%) have confidence in the effectiveness of transplants. All the students stated that in case of their own need or that of a family member, they would authorise the transplant. However, 12.8% of students state that they would refuse the removal of organs from a family member diagnosed with brain death. Moreover, 41.3% of students believe that organ procurement can be refused if seen by relatives as a form of mutilation. Despite the legislative protection on that matter, 20.0% of students say that an illegal organ trade is possible in Italy, 70.6% of students correctly reject it, and a small percentage is unaware of it (9.4%). Almost 95% of nursing students recognise the role of healthcare workers in positively influencing the number of donations (Table 2). In all the items related to attitudes toward organ donation, any statistical significance differences between the students in second year and third year are shown.

The nursing students’ willingness to permit organ procurement from family members in a brain death state (item 9)” revealed several significant associations. This factor was positively correlated with being atheist or agnostic (χ^2^ = 7.235; *p* = 0.022), being in the third year of study (χ^2^ = 4.282; *p* = 0.039) and having positive self-knowledge (χ^2^ = 8.061; *p* = 0.005) (Table 3).

### 3.4. Experience, Source of Information and Proposed Strategies for Transplantation

About 40% of students had the opportunity to meet and/or take care of patients awaiting transplant or who had already received a transplant, some of them for family experiences or acquaintances (15.8%) and others during the internship (25.5%). The main sources of information regarding the subject were advertising campaigns (50.6%) and their university (36.6%), while other sources, such as general practitioners, public offices, etc., were less frequently mentioned. The students’ main proposed strategies to raise the population’s awareness were the involvement of health professionals in secondary schools and universities (53.2%), the use of advertising campaigns (25.5%) and the use of the internet (10.2%), while other proposals were less frequently mentioned by the students (Table 2).

## 4. Discussion

The main purpose of this study was to evaluate nursing students’ knowledge and attitudes regarding organ donation and transplantation. Study participants were mainly female, reflecting the gender of the nursing profession, which is comparable to other previous studies [1,27,35]. The response rate of 67.3% appears to be in line with those of other Italian studies on the topic, which range from 50.0 to 91.9% [1,27,35,36].

### 4.1. Knowledge About Organ Donation, Transplantation and Brain Death

Nursing students’ self-assessed knowledge about transplantation and brain death was found to be suboptimal since less than half of the students declared that they have enough information on transplantation. However, the proportion of students with positive self-assessed knowledge in our sample was higher compared to a previous monocentric study using the same tool (40.4% vs. 25.3%) [27]. In addition, a statistically significant higher percentage of second-year nursing students reported lacking sufficient information compared to third-year students; these data appear to be in line with the literature that identified an influence of educational training received during the academic period on knowledge [27,36,38,39,40].

Although most of the students were aware that ‘brain death’ and’ cardiac death’ did not have the same meaning, almost a quarter of students were unable to correctly define brain death. This lack of clarity was confirmed by the almost 12% of students who replied that returning to life was possible after experiencing brain death. These alarming results are consistent with the previous study of Giusti et al. [27] that found a percentage of 11% for this item. In addition, Martínez-Alarcón et al. [38] found that 70% of students claimed to understand the concept of brain death, while 27% expressed uncertainty and 3% believed that a person with brain death could recover and live a normal life. Better results, on the other hand, were observed in a study conducted in Poland with medical and nursing students, where when analysing self-perceived knowledge of the definition of brain death, it was recorded that approximately 90% of the sample answered correctly [41].

Several additional gaps were identified regarding scientific knowledge, particularly that a fifth of nursing students’ were not aware of the increased workload healthcare workers face when caring for a patient in a state of brain death; however, third-year students demonstrated significantly higher awareness about this item. These findings suggest that students’ knowledge about transplants and brain death tends to improve through their academic career. This could be due to the deeper theoretical preparation from third-year students, as well as to the clinical experience gained given their higher chance of participating in internships in critical departments. In fact, these types of settings represent opportunities for encountering situations related to ethics dilemmas about donations and transplants [42]. Finally, the students also showed themselves not to be fully aware about the factors and circumstances that could alter the state of brain death.

### 4.2. Attitudes to Organ Donation

Students showed an overall positive attitude toward organ donation and transplantation; in fact, most of them stated that in case of need, they would authorise this procedure for themselves or for a family member in a brain death state; despite this, about 13% would not authorise organ procurement from a relative. This could be linked to the fact that a fifth of them believed there is a ‘organ trade’ (black market) in the country, while almost 60% viewed transplantation as mutilation.

About 13% of the sample would not authorise donation or transplantation from a family member in a brain dead state. This could be explained by the fact that almost 59% of students reported that the procurement procedure could be considered a form of mutilation by the relatives of the patient. It seems that students have doubts about the existence of a possible illegal organ trade in our country, despite the protections offered by current legislation [27]. Almost all the participants agreed on the idea that health workers are important for increasing and sensitising the population to organ donation, as shown in previous studies [27,43,44]. All students would accept transplantation as a therapy (both for their own needs and for those of family members), while in other sources, the same figure stood at just over 50% [35,45]. In the event of a family member being in a situation of brain death, most of the students would be inclined to take the sample; if, instead, the family were involved in the decision, the positive attitude would decrease; this could probably be related to the procedure being seen as an act of mutilation of the deceased’s body.

From the literature, the data about the attitudes of Italian nursing students on organ donation are contrasting, ranging from a negative attitude [35] to a positive one [1,11,27]. In our sample, a positive attitude toward organ transplantation was found, albeit with a reduction in this attitude when families are involved [14,27,30,46,47].

The analysis of nursing students’ willingness to permit organ procurement from family members in a brain death state offered insight into several factors that appear to influence students’ attitudes toward organ donation in this specific context. The results highlighted key associations that can help us to better understand the complexities of this decision-making process related to religious beliefs, year of study and the self-knowledge of the student.

The association between nursing students’ willingness to consider organ procurement from family members in a brain death state and their religious beliefs aligns with previous research on the role of religion in shaping attitudes toward organ donation. Religious beliefs are known to significantly influence perspectives on organ donation and transplantation [48,49,50]. In our study, students who identified as atheist or agnostic were more likely to be positive about organ donation, whereas those who were religious were less inclined to support it. This finding is consistent with the broader literature, which suggests that people who believe in God or an afterlife and those who adhere to religious values emphasising bodily integrity after death (such as Confucianism or Buddhism), are generally less likely to endorse organ donation [26,46,48,51]. These religious views often relate to concerns about respecting the body or the connection between bodily integrity and respect for ancestors or nature. However, it is important to note that the majority of students in our study were Catholic, which limited our ability to draw definitive conclusions regarding the influence of other religious beliefs on organ donation attitudes. Despite this, the findings contribute to the existing literature by reinforcing the notion that religious beliefs play a crucial role in shaping attitudes toward organ procurement.

Year of study and self-knowledge were also positively associated with nursing students’ willingness for organ procurement from family members in a brain death state. Third-year students demonstrated a significantly greater willingness to authorise the removal of organs from a family member in a state of brain death compared to second-year students, possibly reflecting higher levels of knowledge and maturity. These results are consistent with the literature that showed that year of study is significantly associated with a commitment to organ donation [36,40,52]. This indicates that as students progress further in their education, their attitudes and perspectives on organ donation may evolve, highlighting the potential impact of continuous education and engagement in shaping these views. Finally, students with positive self-knowledge were more likely to consider organ procurement, possibly due to greater confidence in handling complex ethical issues, as found in previous studies [14,15,53,54]. These findings highlight how factors like religious belief, academic experience and self-awareness shape nursing students’ perspectives on organ donation. Studies have shown that nursing students’ knowledge and attitudes toward organ donation are influenced by various factors, including religious beliefs, cultural influences and personal experiences [14,15,55,56]. These findings suggest that a combination of demographic, educational and personal factors can impact nursing students’ decisions regarding organ donation.

### 4.3. Experience, Source of Information and Proposed Strategies

Most of the students stated that they have not had the opportunity to meet patients who had already undergone transplantation or are on the transplant list, as already described in a previous study [1]. Just over three-quarters of the participants stated that they learned about organ donation through advertising campaigns and/or had received relevant information at school or university. Only a minority of the participants claimed to have received information directly from the organ donor centre; two students claimed to be donors. The students believe that among the strategies required for raising the population’s awareness, the most suitable were speeches given by health professionals in secondary schools and universities and the use of advertising campaigns, the fourth and second options, respectively, chosen in the study by Giusti et al. [27]. In light of current technological advances and in order to facilitate access to the general population, in future studies, it would be interesting to evaluate the effectiveness of virtual active learning methodologies as a strategy to increase awareness for donation, as they have proven to be as effective as face-to-face active learning methodologies [57]. In addition, we suggest designing and validating strategies to increase health profession students’ education, awareness and adherence to donation. For example, in patients with chronic diseases, such as cardiovascular disease, diabetes mellitus and hypertension, they have proven to be valuable elements in improving patient education about their diseases and adherence to treatment [58,59].

### 4.4. Limitations

This study has several limitations. First, although conducted at two Italian universities (in the North and Centre of Italy), we were unable to enrol nursing students from the South of Italy, and the sample was not derived using a randomised multi-stage sampling technique. Furthermore, the small sample size may have affected the significance of the results, which reduces the possibility of generalising the results to all Italian students. It is recommended to repeat the study with a larger sample, including more universities from all of Italy, to validate this hypothesis. Second, while voluntary participation was a key component of this study, this may have introduced selection bias, as those who chose to participate might not fully represent the general nursing student population. To mitigate this, multiple reminders were sent to encourage participation, and the participant pool was expanded to include students from two universities in distinct regions of Italy. This strategy helped to ensure greater diversity and reduce potential bias in the sample. Third, the data were collected using a self-administered questionnaire, so, although we tried to limit problems, evaluation and desirability bias may be present. However, the variables analysed in this study relate to students’ perceptions of their knowledge and attitudes about organ donation and transplantation. Therefore, data collection using self-reported measures was appropriate for perception data. Finally, this study was conducted during the period when the COVID-19 pandemic was ongoing. The pandemic may have influenced the attitudes and perceptions of the students, as public focus shifted toward immediate health concerns and the strain on healthcare systems. The potential impact of COVID-19 on these findings should be considered when interpreting the results, and further research could explore how the pandemic has affected public attitudes toward organ donation.

### 4.5. Future Directions

Future studies could explore and identify additional predictors of knowledge and attitudes toward organ donation among nursing students, building on the associations already investigated in the present study. Further research could examine the effectiveness of different teaching strategies, such as role play and simulation, in improving understanding and attitudes about organ donation. Additionally, exploring the impact of nursing education on students’ ability to collaborate with multidisciplinary teams in organ donation scenarios would provide valuable insights.

### 4.6. Concluding Remarks: Strategies for Raising Awareness and Promoting Organ Donation

To address the chronic shortage of organ donors, it is crucial to raise awareness, beginning with students. This study suggests that improving knowledge and attitudes among nursing students can help to alleviate this issue and save lives in the future. Including mandatory university courses on organ donation, incorporating recommended teaching strategies such as role play and simulation, would provide a strong foundation and build nursing students’ confidence in dealing with this often-neglected topic. Moreover, empowering nurses to teach middle and high school students about organ donation could further spread awareness. The introduction of school nurses, as seen in countries like the United Kingdom, could also be explored in other regions to create an educational bridge between schools, homes and communities. Health policymakers could consider integrating these roles to promote organ donation awareness on a broader scale.

## 5. Conclusions

In our current study, it was found that nursing students’ knowledge appears to be suboptimal: more than half of the students do not believe that they have sufficient information on this topic, with overall better knowledge and attitudes in third-year students in comparison to second-year students. In contrast, attitudes about organ donation appear adequate. This study’s results may help to identify trends in students’ knowledge and attitudes about organ donation and potentially inform educational interventions aimed at raising awareness and encouraging organ donation. Religion, year of study and self-assessed knowledge have been identified as factors influencing nursing students’ willingness to authorise organ procurement from family members in a state of brain death. Further studies are needed to explore the relationship between knowledge, attitudes and other potential predictors for nursing students.

## Figures and Tables

**Table 1:** Socio-demographic characteristics of participants (*n* = 235).

Variable	*n*/%	Mean (SD)/ Median (IQR)
**Age**		23.5 (4.83)/22.0 (21–24)
**Sex**		
Female	199/84.7	
Male	36/15.3	
**High school**		
Scientific	88/37.5	
Technical	61/25.9	
Classical	24/10.2	
Professional	20/8.5	
Other	42/17.9	
**Religion**		
Catholic	126/53.6	
Atheist/Agnostic	90/38.3	
Other religions	19/8.1	
**University site**		
Northern Italy	151/64.3	
Central Italy	84/35.7	
**Academic year**		
Second		
In progress	99/99.0	
Out of course	1/1.0	
Third		
In progress	129/95.6	
Out of course	6/4.4	

IQR: Interquartile range; SD: standard deviation.

**Table 2:** Knowledge and attitudes toward organ donation and transplantation in nursing students stratified by academic year.

Fields/Items			Total (*n* = 235)		Second Year (*n* = 100)		Third Year (*n* = 135)	
			***n***	**%**	***n***	**%**	***n***	**%**
***Self-assessment of knowledge acquired***		**Positive/Correct**						
	1. Do you think that you have sufficient information about transplantation and brain death? *	Yes	95	40.4	31	31.0	64	47.4
	2. Are there laws that regulate these themes?	Yes	230	97.9	98	98.0	132	97.8
***Scientific knowledge***		**Positive/Correct**						
	3. What is ‘brain death’? ^§^	Correct	177	75.3	72	72.0	105	77.8
		Incorrect a	48	20.4	22	22.0	26	19.3
		Incorrect b	10	4.3	6	6.0	4	3.0
	4. Do ‘brain death’ and ‘cardiac death’ have the same meaning?	No	231	98.3	97	97.0	134	99.3
	5. Is it possible for a person to come back to life after being diagnosed with brain death?	No	207	88.1	85	85.0	122	90.4
	6. Do you think that a patient in a state of brain death requires a greater workload for healthcare workers? ^†^	Yes	184	78.3	70	70.0	114	84.4
	7. Is it possible that some circumstances (drug treatment, drug addiction) alter the clinical conditions for the diagnosis of brain death?	Yes	182	77.5	79	79.0	103	76.3
***Attitudes, experiences and personal opinions on donation, organ procurement and transplantation***		**Positive**						
	8. Do you believe in the *effectiveness of transplants*?	Yes	234	99.6	100	100.0	134	99.3
	9. Would you *authorise organ procurement from your family members in a ‘brain death’ state*? ^‡^	Yes	205	87.2	82	82.0	123	91.1
	10. Would you *allow organ transplant as a therapy for you or your family members*, if necessary?	Yes	235	100.0	100	100.0	135	100.0
	11. Do you think that *organ procurement could be refused by relatives as it can be considered a form of mutilation*?	Yes	138	58.7	55	55.0	83	61.48
	12. Despite legislative protection, do you think that a *‘trade’ in organs exists* in our country?	Yes	47	20.0	23	23.0	24	17.8
		No	22	9.4	8	8.0	14	10.4
		I don’t know	166	70.6	69	69.0	97	71.9
	13. Do you think that *healthcare workers are important for increasing transplants*?	Yes	223	94.9	94	94.0	129	95.6
***Experience and lived experiences***		***Responses***						
	14. Have you already had *experience* with clients in need of or who underwent to a transplant?	Yes, acquaintances or family members	37	15.8	14	14.0	23	17.0
		Yes, during the internship	60	25.5	10	10.0	50	37.0
		No	138	58.7	76	76.0	62	45.9
***Information and proposals***		***Responses***						
	15. From which *main ‘source’* did you find information on the subject?	Information advertising campaigns (newspapers, radio, internet)	119	50.6	54	54.0	65	48.2
		School, university (internship)	86	36.6	32	32.0	54	40.0
		Family	9	3.8	5	5.0	4	3.0
		General practitioner	7	2.9	4	4.0	3	2.2
		Public offices (e.g., Municipal Registry)	7	2.9	2	2.0	5	3.7
		Organ and tissue donation centres	7	2.9	3	3.0	4	3.0
	16. What should be the *main strategy* to raise awareness among the population on this issue?	Health professionals in upper-secondary schools and universities	125	53.2	53	53.0	72	53.3
		Use of advertising campaigns (TV, documentaries, testimonials, commercials, brochures)	60	25.5	24	24.0	36	26.7
		Use of the internet (blogs, forums, social networks, e-mails)	24	10.2	12	12.0	12	8.9
		Diffusion of movies and short movies	17	7.2	7	7.0	10	7.4
		Explanation of pros and cons through the participation of Very Important People	5	2.1	1	1.0	4	3.0
		Show people’s suffering	4	1.8	3	3.0	1	0.7

* *p* = 0.011; ^†^ *p* = 0.008; ^‡^ *p* = 0.039, ^§^ Answers to Item 3—Correct: The irreversible cessation of all brain functions but not the arrest of other biological activities; Incorrect a: The irreversible cessation of all brain functions resulting from the arrest of any other biological activity; Incorrect b: A state of irreversible coma.

**Table 3:** Factors associated with nursing students’ willingness to permit organ donation (*n* = 235).

Variables	Willingness for Organ Procurement from Your Family Members in a ‘Brain Death’ State (Item 9)				
	**Yes**		**No**		**c^2^/U Test;** ***p*-Value**
	***n***	**%**	***n***	**%**	
**Gender**					
Female	176	88.4	23	11.6	1.703
Male	29	80.6	7	19.4	0.185
**Religion beliefs**					
Catholic	109	86.5	17	13.5	
Atheist/Agnostic	83	92.2	7	7.8	7.235
Other religions	13	68.4	6	31.6	**0.022**
**University site**					
Northern Italy	131	86.8	20	13.2	0.087
Central Italy	74	88.1	10	11.9	0.768
**Academic year**					
Second	82	82.0	18	18.0	4.282
Third	132	91.1	12	8.9	**0.039**
**High school**					
Scientific	76	86.4	12	13.6	
Technical	51	83.6	10	16.4	2.001
Classical	21	87.5	3	12.5	0.744 *
Professional	18	90.0	2	10.0	
Other	39	92.9	3	7.1	
**Age (years)**					
Average (SD)	23.55	(4.816)	23.23	(4.960)	2625
					0.190 **
**Self-knowledge (Item 1)**					
Positive self-knowledge	90	94.7	5	5.3	8.061
Negative self-knowledge	115	82.1	25	17.9	**0.005**

* Chi-square test, ** Mann–Whitney U test. Bold values denote statistical significance at the *p* < 0.05 level.

## Data Availability

The raw data supporting the conclusions of this article will be made available by the authors on request.

## Public Involvement Statement

No public involvement in any aspect of this research.

## Guidelines and Standards Statement

This manuscript was drafted by following the Strengthening the Reporting of Observational Studies in Epidemiology (STROBE) [37] for cross-sectional research.
